# Differences between complex epithelial neoplasms of the ovary and high-grade serous ovarian cancer: a retrospective observational cohort study

**DOI:** 10.1186/s13048-022-01063-4

**Published:** 2022-12-01

**Authors:** Xiaoxue Li, Yiling Ding, Yang Liu, Mengyuan Yang

**Affiliations:** Department of Obstetrics and Gynecology, The Second Xiangya Hospital of Central South University, Changsha, 410011 China

**Keywords:** Complex epithelial neoplasms of the ovary, High-grade serous ovarian cancer, Adenosquamous carcinoma, Adenocarcinoma with metaplasia, Overall survival, Cancer-specific survival, SEER database, Propensity score matching

## Abstract

**Background:**

Complex epithelial neoplasms of the ovary (CENO), an uncommon pathological histotype in ovarian cancer, comprises adenosquamous carcinoma and adenocarcinoma with metaplasia. Owing to the rarity of relevant reports, there are currently no statistics on outcomes based on large samples. Meanwhile high-grade serous ovarian cancer (HGSOC) is the most common histotype in ovarian cancer which has a recognized first-line treatment regimen and poor prognosis. Thus, we aimed to determine the characteristics, prognosis, and independent predictors of survival for CENO, compare them with those of HGSOC and construct prognostic predictive models and nomograms.

**Methods:**

We used the Surveillance, Epidemiology, and End Results (SEER) database to determine patients diagnosed with CENO or HGSOC from 2000 to 2017. Clinical, demographic, and treatment characteristics were compared between these groups. Propensity score matching, Cox risk regression analysis, Kaplan–Meier survival curves, and the Least Absolute Shrinkage and Selection Operator regression analysis were employed for analyzing the data.

**Results:**

Here, 31,567 patients with HGSOC and 216 patients with CENO between 2000 and 2017 in the SEER database were enrolled. Age < 57 years, unmarried, and early-stage diseases were more common in patients with CENO than in those with HGSOC. Women with CENO were less likely to receive adjuvant chemotherapy (65.7% vs. 79.4%) but more likely to receive radiotherapy (6.0% vs. 0.8%; both *p* < 0.001) than those with HGSOC. Year of diagnosis, surgery status, number of primary tumors, grade, and FIGO stage were independent prognostic factors for overall and cancer-specific survival in CENO. Overall survival rates were significantly lower for CENO than for more malignant HGSOC.

**Conclusions:**

In summary, CENO was rare in ovarian cancer, while the year of diagnosis, surgery status, number of primary tumors, grade, and FIGO stage were independent prognostic factors. Compared with other common malignant ovarian tumors, CENO had a poor prognosis. Prognostic predictive models and nomograms had been determined to predict the individual survival rates of patients with CENO. These methods could improve evaluations of survival and therapeutic decisions for patients.

## Introduction

Ovarian cancer, one of the most prevalent cancers in women, is a common cause of death from gynecologic tumors [1, 2]. Epithelial neoplasms, which are identified as tumors that develop within the epithelium and occupy 90% of ovarian cancer pathological types, are classified into high-grade serous ovarian carcinoma (HGSOC), mucinous tumors, endometrioid tumors, and clear cell tumors in the ovary [3, 4]. Of the various subtypes, HGSOC is the most common and deadliest in ovarian cancer, characterized by sized papillary architecture with slit-like spaces and high-grade nuclei. Patients with HGSOC are usually diagnosed at an advanced stage since there are only a few early-specific symptoms. The initial response to first-line platinum-based chemotherapy in patients with HGSOC is typically good; however, most of them eventually develop resistance to the treatment and, consequently, relapse [5–7].

Complex epithelial neoplasms of the ovary (CENO), a pathologically broad histotype in epithelial ovarian cancer, comprises two subtypes, namely adenosquamous carcinoma (adenosquamous and epithelial-myoepithelial carcinoma) and adenocarcinoma with metaplasia (adenocarcinoma with squamous metaplasia and adenocarcinoma with neuroendocrine differentiation, metaplastic carcinoma, and hepatoid adenocarcinoma) according to the World Health Organization guidelines [8–10]. CENO, composed of adenocarcinoma and other epithelial cells transformed from gland cells, is extremely rare in ovarian cancer. Presently, studies on these pathological types of ovarian cancer exist only as case reports. Adenosquamous carcinoma, which accounts for < 1% of all ovarian malignancies, is characterized as a carcinoma showing components of both adenocarcinoma and squamous cell carcinoma, with each comprising at least 10% of the tumor [11, 12]. By far, records of no more than 30 cases of adenosquamous carcinomas of ovaries are available on PubMed [12–15]. In these case reports, this subtype of ovarian cancer is featured as a highly malignant disease; metastasis and recurrence are prone to occur even in the early stages. Epithelial-myoepithelial carcinoma is an uncommon low-grade gland carcinoma characterized by biphasic tubular structures composed of inner eosinophilic ductal cells and outer clear myoepithelial cells, pathologically classified as adenosquamous carcinoma [16]. Adenocarcinoma with squamous metaplasia and neuroendocrine differentiation is characterized as adenocarcinoma of the ovary showing squamous differentiation [17, 18] and differentiated neuroendocrine cells scattered in the form of a single cell or cell nests [19], respectively. Metaplastic carcinoma is a unique mixed-tumor whose glandular component may be partially or completely replaced by a non-glandular component such as squamous, spindle, and chondroid cells [20]. Hepatoid adenocarcinoma is a tumor with a phenotype resembling that of hepatocellular carcinoma, which secretes alpha-fetoprotein (AFP) [21]. Only a few cases of these aforementioned pathological types in ovarian cancer have been reported on PubMed. Owing to the extremely low incidence of CENO and scarcity of relevant reports in the literature, recognized first-line treatment strategy and statistics related to prognosis in large samples for CENO or each pathological subtype in CENO are currently lacking.

In this study, we aimed to determine the clinical characteristics, treatment strategies, and prognosis of CENO based on a large-scale sample. We further sought to analyze the overall survival (OS) and cancer-specific survival (CSS) rates using data from the Surveillance, Epidemiology, and End Results (SEER) database. Moreover, we intended to employ these data for determining the prognostic factors of CENO. HGSOC is the most common subtype of ovarian cancer associated with poor prognosis and being diagnosed at an advanced stage. No population-based study comparing the differences between CENO and HGSOC exists thus far. Therefore, we further compared the clinical characteristics and prognosis of patients between those with CENO and HGSOC to provide a reference for clinical treatment and a novel prognosis prediction method.

## Results

### Demographic characteristics

**Fig. 1:**
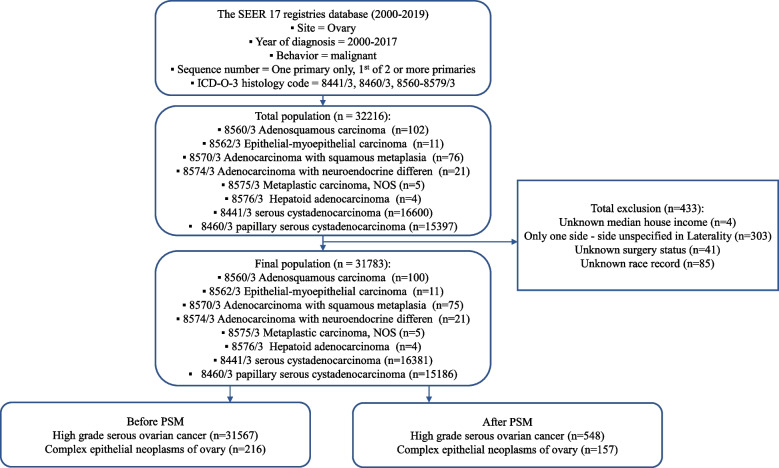
Schematic illustration of the study workflow

**Fig. 2:**
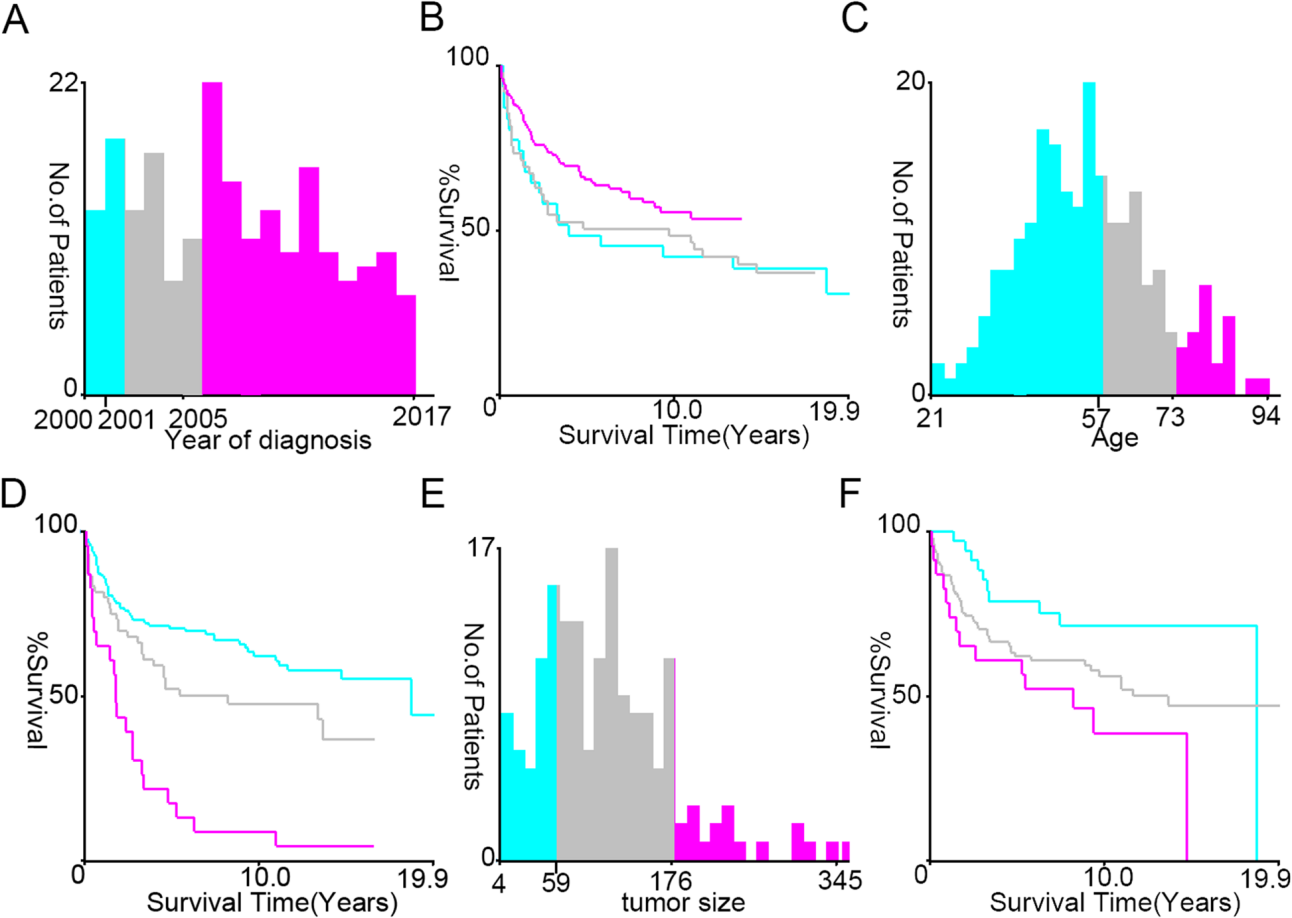
Identification of optimal cut-off values for the various clinical characteristics using X-tile software analysis. Year of diagnosis: (**a**) best cut-off value for the year of diagnosis and (**b**) survival curves for different years of diagnosis. Age: (**c**) best cut-off value for age and (**d**) survival curves for different ages. Tumor size: (**e**) best cut-off value for tumor size and (**f**) survival curves for different tumor sizes. Based on overall survival, the optimal minimum and maximum cut-off values for the year of diagnosis are 2001 and 2005, respectively, while those for age at diagnosis and tumor sizes are 57 and 73 years and 59 and 176 mm, respectively

The CONSORT diagram for the study selection is depicted in Fig. 1. In this study, 31,567 patients with HGSOC and 216 patients with CENO between 2000 and 2017 in the SEER database were enrolled. The differences in four basic demographic characteristics were assessed between patients with HGSOC and patients with CENO. Here, the optimal cut-off value of continuous variables, such as year of diagnosis, age, and tumor size, were assessed using the X-tile software (Yale University, USA), followed by the conversion of these variables into categorical variables. The best minimum and maximum cut-off values for the year of diagnosis were 2001 and 2006, respectively, while those for tumor sizes and age at diagnosis were 59 and 176 mm and 57 and 73 years, respectively (Fig. 2). Patients with HGSOC were older than those with CENO (mean: 63.0 years vs. 48.0 years, *p* < 0.001). Regarding race, 85.3% of patients with HGSOC were white compared with 79.6% of patients with CENO (*p* = 0.012). Regarding marital status at diagnosis, 80.7% of patients were married, divorced, separated, or widowed when they were diagnosed with HGSOC compared with 70.9% of patients with CENO (*p* = 0.001). No difference was observed in terms of year of diagnosis and median household income between patients with HGSOC and patients with CENO (Table 1).

**Table 1:** Demographic and clinical characteristics comparing CENO and HGSOC (pre-matching and pos-matching)

Subject	Before propensity score matching		*P*-value	After propensity score matching		*P*-value
Characteristic	HGSOC	CENO		HGSOC	CENO	
	N(%)	N(%)		N(%)	N(%)	
All	31,567	216		548	157	
**Year of diagnosis**						
2000–2001	3058 (9.7)	31 (14.4)	0.038	86 (15.7)	25 (15.9)	0.904
2002–2005	6108 (19.3)	46 (21.3)		124 (22.6)	38 (24.2)	
2006–2017	22,401 (71.0)	139 (64.4)		338 (61.7)	94 (59.9)	
**Laterality**						
Bilateral	16,277 (51.6)	51 (23.6)	< 0.001	175 (31.9)	42 (26.8)	0.609
Left	5973 (18.9)	82 (38.0)		179 (32.7)	57 (36.3)	
Paired site, but no information concerning laterality	3010 (9.5)	11 (5.1)		32 (5.8)	11 (7.0)	
Right	6307 (20.0)	72 (33.3)		162 (29.6)	47 (29.9)	
**Surgery status**						
No surgery	3356 (10.6)	16 (7.4)	0.155	52 (9.5)	15 (9.6)	1
Surgery	28,211 (89.4)	200 (92.6)		496 (90.5)	142 (90.4)	
**Radiation therapy**						
No radiation	31,300 (99.2)	203 (94.0)	< 0.001	525 (95.8)	145 (92.4)	0.123
Radiation	267 (0.8)	13 (6.0)		23 (4.2)	12 (7.6)	
**Chemotherapy status**						
Chemotherapy	25,056 (79.4)	142 (65.7)	< 0.001	358 (65.3)	99 (63.1)	0.667
No chemotherapy/Unknown	6511 (20.6)	74 (34.3)		190 (34.7)	58 (36.9)	
**Number of primary tumors**						
1st of 2 or more primaries	2435 (7.7)	49 (22.7)	< 0.001	70 (12.8)	22 (14.0)	0.786
One primary only	29,132 (92.3)	167 (77.3)		478 (87.2)	135 (86.0)	
**Race**						
Black	2145 (6.8)	15 (6.9)	0.012	40 (7.3)	13 (8.3)	0.909
Other (American Indian/AK Native, Asian/Pacific Islander)	2505 (7.9)	29 (13.4)		73 (13.3)	20 (12.7)	
White	26,917 (85.3)	172 (79.6)		435 (79.4)	124 (79.0)	
**Marital status at diagnosis**						
DSW	8665 (27.4)	52 (24.1)	0.001	152 (27.7)	40 (25.5)	0.941
Married	16,818 (53.3)	101 (46.8)		253 (46.2)	74 (47.1)	
Single	4926 (15.6)	56 (25.9)		122 (22.3)	36 (22.9)	
Unknown	1158 (3.7)	7 (3.2)		21 (3.8)	7 (4.5)	
**Median household income**						
< 50,000	3484 (11.0)	16 (7.4)	0.148	50 (9.1)	14 (8.9)	0.854
>=70,000	13,582 (43.0)	90 (41.7)		236 (43.1)	64 (40.8)	
50,000-69,999	14,501 (45.9)	110 (50.9)		262 (47.8)	79 (50.3)	
**Age**						
<=57	11,147 (35.3)	133 (61.6)	< 0.001	304 (55.5)	88 (56.1)	0.986
>=74	6712 (21.3)	23 (10.6)		83 (15.1)	23 (14.6)	
58–73	13,708 (43.4)	60 (27.8)		161 (29.4)	46 (29.3)	
**Grade**						
I/II	4476 (14.2)	102 (47.2)	< 0.001	188 (34.3)	58 (36.9)	0.606
III/IV	20,190 (64.0)	77 (35.6)		262 (47.8)	68 (43.3)	
Unknown	6901 (21.9)	37 (17.1)		98 (17.9)	31 (19.7)	
**Seer stage**						
Distant	24,533 (77.7)	76 (35.2)	< 0.001	273 (49.8)	74 (47.1)	0.909
Localized	1671 (5.3)	62 (28.7)		104 (19.0)	33 (21.0)	
Regional	4880 (15.5)	74 (34.3)		155 (28.3)	46 (29.3)	
Unknown	483 (1.5)	4 (1.9)		16 (2.9)	4 (2.5)	
**Stage**						
I	2732 (8.7)	88 (40.7)	< 0.001	154 (28.1)	42 (26.8)	0.917
II	2243 (7.1)	34 (15.7)		70 (12.8)	23 (14.6)	
III	15,381 (48.7)	43 (19.9)		157 (28.6)	42 (26.8)	
IV	8980 (28.4)	34 (15.7)		103 (18.8)	33 (21.0)	
Unknown	2231 (7.1)	17 (7.9)		64 (11.7)	17 (10.8)	
**tumor size**						
<=59	6635 (21.0)	34 (15.7)	< 0.001	99 (18.1)	27 (17.2)	0.782
> = 177	1387 (4.4)	23 (10.6)		33 (6.0)	13 (8.3)	
60–176	11,894 (37.7)	106 (49.1)		248 (45.3)	71 (45.2)	
Unknown	11,651 (36.9)	53 (24.5)		168 (30.7)	46 (29.3)	
**Lymph nodes status**						
negative	7521 (23.8)	26 (12.0)	< 0.001	91 (16.6)	26 (16.6)	0.855
positive	7042 (22.3)	94 (43.5)		176 (32.1)	54 (34.4)	
Unknown	17,004 (53.9)	96 (44.4)		281 (51.3)	77 (49.0)	
**CA125**						
negative	1266 (4.0)	105 (48.6)	< 0.001	165 (30.1)	53 (33.8)	0.383
positive	19,276 (61.1)	24 (11.1)		109 (19.9)	24 (15.3)	
Unknown	11,025 (34.9)	87 (40.3)		274 (50.0)	80 (51.0)	

*CENO* complex epithelial neoplasms of the ovary

*HGSOC* high-grade serous ovarian cancer

### Clinicopathological characteristics and treatment

Over half of the HGSOC cases (51.6%) between 2000 and 2017 in the SEER database were bilateral, whereas only 23.6% of the CENO cases were bilateral (*p* < 0.001). HGSOCs were more likely to be advanced-stage disease (FIGO stage III/IV: 77.1% vs. 35.6%; grade III/IV: 64.0% vs. 35.6%) and had a smaller tumor size (< 59 mm: 21.0% vs. 15.7%) compared to CENO (both, *p* < 0.001). Patients with HGSOC were more likely to have only one primary tumor (92.3% vs. 77.3%), positive cancer antigen 125 (CA125) levels (61.1% vs. 11.1%), and lower positive lymph nodes status (22.3% vs. 43.5%) at the time of surgery (all, *p* < 0.001).

In total, 89.4% of patients with HGSOC underwent surgery compared with 92.6% of patients with CENO who underwent surgery (*p* = 0.155). Patients with HGSOC were more likely to receive chemotherapy (79.4% vs. 65.7%, *p* < 0.001) and less likely to receive radiotherapy (0.8% vs. 6.0%, *p* < 0.001) compared with those with CENO (Table 1).

### Survival analysis

**Fig. 3:**
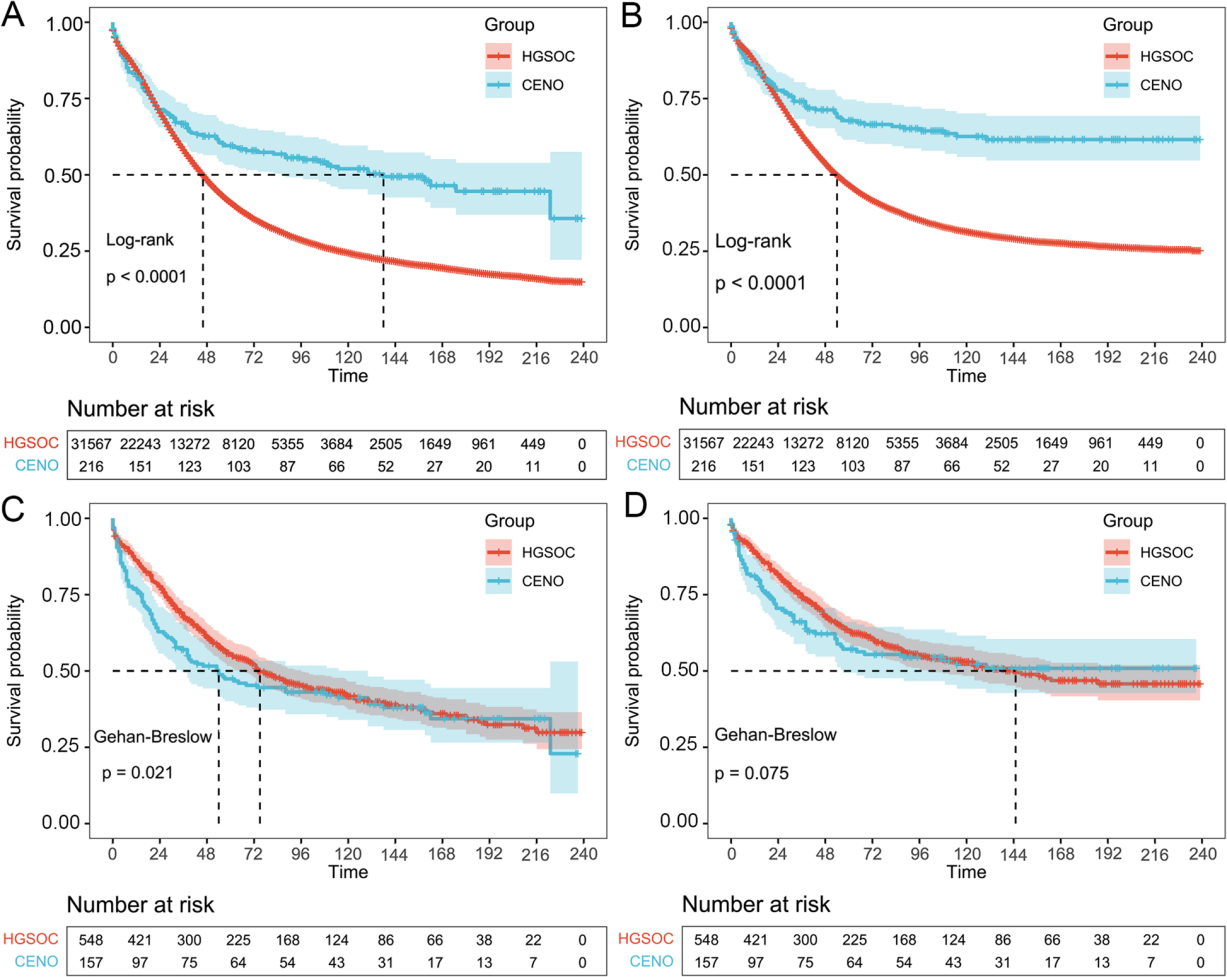
Survival outcomes before and after propensity score matching. **a** Overall survival and (**b**) cancer-specific survival based on patients with HGSOC or CENO before propensity score matching. Log-rank tests are used to generate the *p*-values. **c** Overall survival and (**d**) cancer-specific survival based on patients with HGSOC or CENO after propensity score matching. The Gehan-Breslow tests are used to generate the *p*-values. HGSOC, high-grade serous ovarian cancer; CENO, complex epithelial neoplasms of the ovary

In the SEER program, the median OS and CSS time of patients with HGSOC were 46.0 and 54.0 months, respectively. The 1-, 3-, and 5-year OS rates were 84.3, 58.8, and 44.0%, respectively, while the 1-, 3-, and 5-year CSS rates were 87.2, 65.0, and 52.4%, respectively. The median OS time of patients with CENO was 130.0 months, while the median CSS time was not reached. The 1-, 3-, and 5-year OS rates were 82.4, 67.1, and 60.6%, respectively, while the 1-, 3-, and 5-year CSS rates were 86.1, 75.5, and 70.4%, respectively (P_log-tank_ < 0.001). These results indicate that the survival of patients with CENO was better than those with HGSOC before propensity score matching (PSM) (P_log-tank_ < 0.001, Fig. 3a-b).

After PSM matching, cases of 548 patients with HGSOC and 157 patients with CENO were included in this study. The baseline characteristics between these two groups were well balanced and included in further analysis (Table 1). The median OS and CSS time of patients with HGSOC after PSM matching were 74.0 and 140.0 months, respectively. The 1-, 3-, and 5-year OS rates were 88.1, 68.1, and 57.3%, respectively, while the 1-, 3-, and 5-year CSS rates were 89.2, 75.4, and 66.8%, respectively. For patients with CENO after PSM matching, the median OS time was 53.0 months, while the median CSS time was not reached. The 1-, 3-, and 5-year OS rates were 76.4, 57.3, and 48.4%, respectively, while the 1-, 3-, and 5-year CSS rates were 81.5, 68.2, and 61.2%, respectively. As the survival curves for the cases after PSM crossed over, we employed the Gehan-Breslow test to compare the survival status between patients with HGSOC and patients with CENO. The results determined that the OS of patients with CENO was worse than that of patients with HGSOC (P_Breslow_ = 0.021; Fig. 3c-d).

### Identification of prognostic factors for patients with CENO

To determine the prognostic factors for better disease outcomes, a cohort of 216 patients with CENO in the SEER database was analyzed via univariate and multivariate Cox regression analysis. The results of the univariate regression analysis are presented in Table 2. Age, race, marital status at diagnosis, median household income, tumor size, grade, laterality, SEER stage, FIGO stage, surgery status, CA125 status, and the number of primary tumors were significantly associated with both OS and CSS of CENO (*p* < 0.05), whereas the year of diagnosis was only associated with CSS of CENO (*p* = 0.028). Thereafter, we incorporated these variables into a multivariate Cox regression analysis to determine the independent prognostic factors. The results exhibited that surgery status (surgery vs. no surgery: HR = 0.18, *p* < 0.001), number of primary tumors (only one primary tumor vs. first of ≥2 primary tumors: HR = 2.00, *p* = 0.046), race (other vs. black: HR = 0.35, *p* = 0.043; white vs. black: HR = 0.40, *p* = 0.014), grade (III/IV vs. I/II: HR = 2.14, *p* = 0.043), and FIGO stage (I: reference; II: HR = 6.80, *p* = 0.003; III: HR = 6.49, *p* = 0.012; IV: HR = 6.49, *p* = 0.018) were independent OS prognostic factors for CENO (Table 3). Regarding CSS, the results indicated that year of diagnosis (2006–2017 vs. 2000–2001: HR = 0.44, *p* = 0.044), surgery status (surgery vs. no surgery: HR = 0.27, *p* = 0.011), number of primary tumors (only one primary tumor vs. first of ≥2 primary tumors: HR = 2.99, *p* = 0.03), grade (III/IV vs. I/II: HR = 3.08, *p* = 0.004), and FIGO stage (I: reference; II: HR = 7.89, *p* = 0.024; III: HR = 5.93, *p* = 0.072; IV: HR = 7.49, *p* = 0.055) were independent prognostic factors for CENO (Table 3).

**Table 2:** Univariable Cox Regression for analyzing the associated factors for complex epithelial neoplasms of the ovary

Subject characteristics	Overall survival (OS)		Cancer-specific survival (CSS)	
	HR (95%CI)	*P*-value	HR (95%CI)	*P*-value
**Year of diagnosis**				
2000–2001	Reference		Reference	
2002–2005	0.99(0.55 ~ 1.77)	0.971	0.89(0.46 ~ 1.74)	0.739
2006–2017	0.66 (0.39 ~ 1.12)	0.121	0.51 (0.28 ~ 0.93)	0.028
**Laterality**				
Bilateral	Reference		Reference	
Left	0.61 (0.38 ~ 0.98)	**0.041**	0.54 (0.31 ~ 0.96)	**0.035**
Paired site, but no information concerning laterality	3.92 (1.94 ~ 7.93)	**< 0.001**	3.08 (1.3 ~ 7.29)	**0.01**
Right	0.36 (0.21 ~ 0.63)	**< 0.001**	0.41 (0.22 ~ 0.76)	**0.005**
**Surgery status**				
No surgery	Reference		Reference	
Surgery	0.11 (0.06 ~ 0.19)	**< 0.001**	0.16 (0.08 ~ 0.33)	**< 0.001**
**Radiation therapy**				
No radiation	Reference		Reference	
Radiation	1.04 (0.48 ~ 2.24)	0.924	0.61 (0.19 ~ 1.94)	0.404
**Chemotherapy status**				
Chemotherapy	Reference		Reference	
No chemotherapy/Unknown	1.03 (0.69 ~ 1.55)	0.883	0.86 (0.52 ~ 1.44)	0.573
**Number of primary tumors**				
1st of 2 or more primaries	Reference		Reference	
One primary only	3.28 (1.75 ~ 6.12)	**< 0.001**	5.13 (2.06 ~ 12.72)	**< 0.001**
**Race**				
Black	Reference		Reference	
Other (American Indian/AK Native, Asian/Pacific Islander)	0.3 (0.13 ~ 0.69)	**0.005**	0.32 (0.12 ~ 0.83)	**0.019**
White	0.36 (0.2 ~ 0.66)	**0.001**	0.34 (0.17 ~ 0.69)	**0.003**
**Marital status at diagnosis**				
DSW	Reference		Reference	
Married	0.53 (0.33 ~ 0.84)	**0.006**	0.52 (0.3 ~ 0.91)	**0.021**
Single	0.59 (0.35 ~ 1.01)	0.053	0.68 (0.37 ~ 1.25)	0.217
Unknown	0.71 (0.25 ~ 2)	0.512	0.53 (0.12 ~ 2.24)	0.388
**Median household income**				
< 50,000	Reference		Reference	
>=70,000	0.44 (0.23 ~ 0.84)	**0.013**	0.39 (0.18 ~ 0.85)	**0.018**
50,000-69,999	0.48 (0.26 ~ 0.91)	**0.023**	0.51 (0.25 ~ 1.05)	0.069
**Age**				
<=57	Reference		Reference	
>=74	4.47 (2.68 ~ 7.45)	**< 0.001**	2.92 (1.47 ~ 5.78)	**0.002**
58–73	1.65 (1.06 ~ 2.58)	**0.028**	1.85 (1.11 ~ 3.07)	**0.018**
**Grade**				
I/II	Reference		Reference	
III/IV	3.76 (2.37 ~ 5.98)	**< 0.001**	5.59 (3.05 ~ 10.26)	**< 0.001**
Unknown	3.61 (2.07 ~ 6.28)	**< 0.001**	4.8 (2.34 ~ 9.84)	**< 0.001**
**Seer stage**				
Distant	Reference		Reference	
Localized	0.14 (0.08 ~ 0.25)	**< 0.001**	0.09 (0.04 ~ 0.22)	**< 0.001**
Regional	0.31 (0.19 ~ 0.49)	**< 0.001**	0.31 (0.18 ~ 0.54)	**< 0.001**
Unknown	1.43 (0.45 ~ 4.59)	0.547	1.24 (0.3 ~ 5.14)	0.768
**FIGO Stage**				
I	Reference		Reference	
II	5.14 (2.53 ~ 10.43)	**< 0.001**	7.88 (2.99 ~ 20.76)	**< 0.001**
III	8.59 (4.47 ~ 16.5)	**< 0.001**	11.51 (4.63 ~ 28.61)	**< 0.001**
IV	11.46 (5.89 ~ 22.31)	**< 0.001**	18.46 (7.48 ~ 45.57)	**< 0.001**
Unknown	10.43 (4.82 ~ 22.59)	**< 0.001**	14.74 (5.23 ~ 41.55)	**< 0.001**
**tumor size**				
<=59	Reference		Reference	
>=177	2.63 (1.17 ~ 5.92)	**0.02**	3.23 (1.08 ~ 9.64)	**0.036**
60–176	1.74 (0.88 ~ 3.44)	0.112	2.5 (0.98 ~ 6.4)	0.056
Unknown	2.99 (1.47 ~ 6.08)	**0.002**	4.34 (1.66 ~ 11.4)	**0.003**
**Lymph nodes status**				
negative	Reference		Reference	
positive	1.28 (0.62 ~ 2.65)	0.501	1.14 (0.5 ~ 2.62)	0.755
Unknown	1.8 (0.89 ~ 3.64)	0.105	1.69 (0.75 ~ 3.78)	0.204
**CA125**				
negative	Reference		Reference	
positive	4.19 (2.25 ~ 7.8)	**< 0.001**	5.46 (2.69 ~ 11.11)	**< 0.001**
Unknown	3.93 (2.49 ~ 6.22)	**< 0.001**	4.07 (2.3 ~ 7.18)	**< 0.001**

*HR* Hazard Ratio; *CI* Confidence Interval

Bold means *p* < 0.05

**Table 3:** Multivariable Cox Regression for analyzing the associated factors for complex epithelial neoplasms of the ovary

Subject characteristics	Overall survival (OS)		Cancer-specific survival (CSS)	
	HR (95%CI)	*P*-value	HR (95%CI)	*P*-value
**Year of diagnosis**				
2000–2001			Reference	
2002–2005			0.85 (0.37 ~ 1.99)	0.713
2006–2017			0.44 (0.2 ~ 0.98)	**0.044**
**Laterality**				
Bilateral	Reference		Reference	
Left	0.81 (0.45 ~ 1.45)	0.475	0.89(0.43 ~ 1.82)	0.746
Paired site, but no information concerning laterality	0.74 (0.27 ~ 2.03)	0.554	1.02 (0.31 ~ 3.32)	0.972
Right	0.53 (0.27 ~ 1.05)	0.068	0.75 (0.33 ~ 1.68)	0.481
**Surgery status**				
No surgery	Reference		Reference	
Surgery	0.18 (0.07 ~ 0.41)	**< 0.001**	0.27 (0.1 ~ 0.75)	**0.011**
**Number of primary tumors**				
1st of 2 or more primaries	Reference		Reference	
One primary only	2 (1.01 ~ 3.97)	**0.046**	2.99 (1.11 ~ 8.02)	**0.03**
**Race**				
Black	Reference		Reference	
Other (American Indian/AK Native, Asian/Pacific Islander)	0.35 (0.13 ~ 0.97)	**0.043**	0.58 (0.18 ~ 1.87)	0.362
White	0.4 (0.19 ~ 0.83)	**0.014**	0.43 (0.18 ~ 1.01)	0.053
**Marital status at diagnosis**				
DSW	Reference		Reference	
Married	0.6 (0.34 ~ 1.05)	0.072	0.81 (0.38 ~ 1.71)	0.582
Single	0.83 (0.41 ~ 1.64)	0.585	1.14 (0.47 ~ 2.75)	0.77
Unknown	0.34 (0.08 ~ 1.46)	0.145	0.39 (0.05 ~ 2.79)	0.349
**Median household income**				
< 50,000	Reference		Reference	
>=70,000	1.01 (0.45 ~ 2.23)	0.987	0.69 (0.27 ~ 1.77)	0.44
50,000-69,999	0.96 (0.44 ~ 2.08)	0.916	1.02 (0.41 ~ 2.54)	0.968
**Age**				
<=57	Reference		Reference	
>=74	1.57 (0.79 ~ 3.11)	0.197	0.96 (0.38 ~ 2.41)	0.924
58–73	0.88 (0.49 ~ 1.58)	0.667	1.15 (0.57 ~ 2.34)	0.696
**Grade**				
I/II	Reference		Reference	
III/IV	2.14 (1.21 ~ 3.78)	**0.009**	3.08 (1.45 ~ 6.54)	**0.004**
Unknown	2.1 (1.03 ~ 4.31)	**0.042**	3.48 (1.47 ~ 8.29)	**0.005**
**Seer stage**				
Distant	Reference		Reference	
Localized	2.19 (0.51 ~ 9.37)	0.292	1.67 (0.23 ~ 12.41)	0.615
Regional	1.19 (0.42 ~ 3.43)	0.741	1.17 (0.34 ~ 4.11)	0.801
Unknown	0.97 (0.21 ~ 4.42)	0.973	1.01 (0.16 ~ 6.39)	0.994
**Stage**				
I	Reference		Reference	
II	6.8 (1.89 ~ 24.44)	**0.003**	7.89 (1.32 ~ 47.2)	**0.024**
III	6.35 (1.51 ~ 26.8)	**0.012**	5.93 (0.85 ~ 41.28)	0.072
IV	6.49 (1.39 ~ 30.39)	**0.018**	7.49 (0.96 ~ 58.33)	0.055
Unknown	13.76 (3.65 ~ 51.85)	**< 0.001**	12.25 (1.74 ~ 86)	**0.012**
**tumor size**				
<=59	Reference		Reference	
>=177	2.48 (0.98 ~ 6.28)	0.055	3.07 (0.87 ~ 10.76)	0.08
60–176	1.28 (0.58 ~ 2.81)	0.543	1.36 (0.47 ~ 3.98)	0.57
Unknown	1.23 (0.53 ~ 2.9)	0.63	1.03 (0.32 ~ 3.28)	0.964
**CA125**				
negative	Reference		Reference	
positive	1.55 (0.65 ~ 3.69)	0.319	2.45 (0.84 ~ 7.21)	0.102
Unknown	1.93 (1.03 ~ 3.6)	**0.04**	2.08 (0.89 ~ 4.9)	0.093

*HR* Hazard Ratio; *CI* Confidence Interval

Bold means *p* < 0.05

### Construction of predictive models and nomograms for OS and CSS

**Fig. 4:**
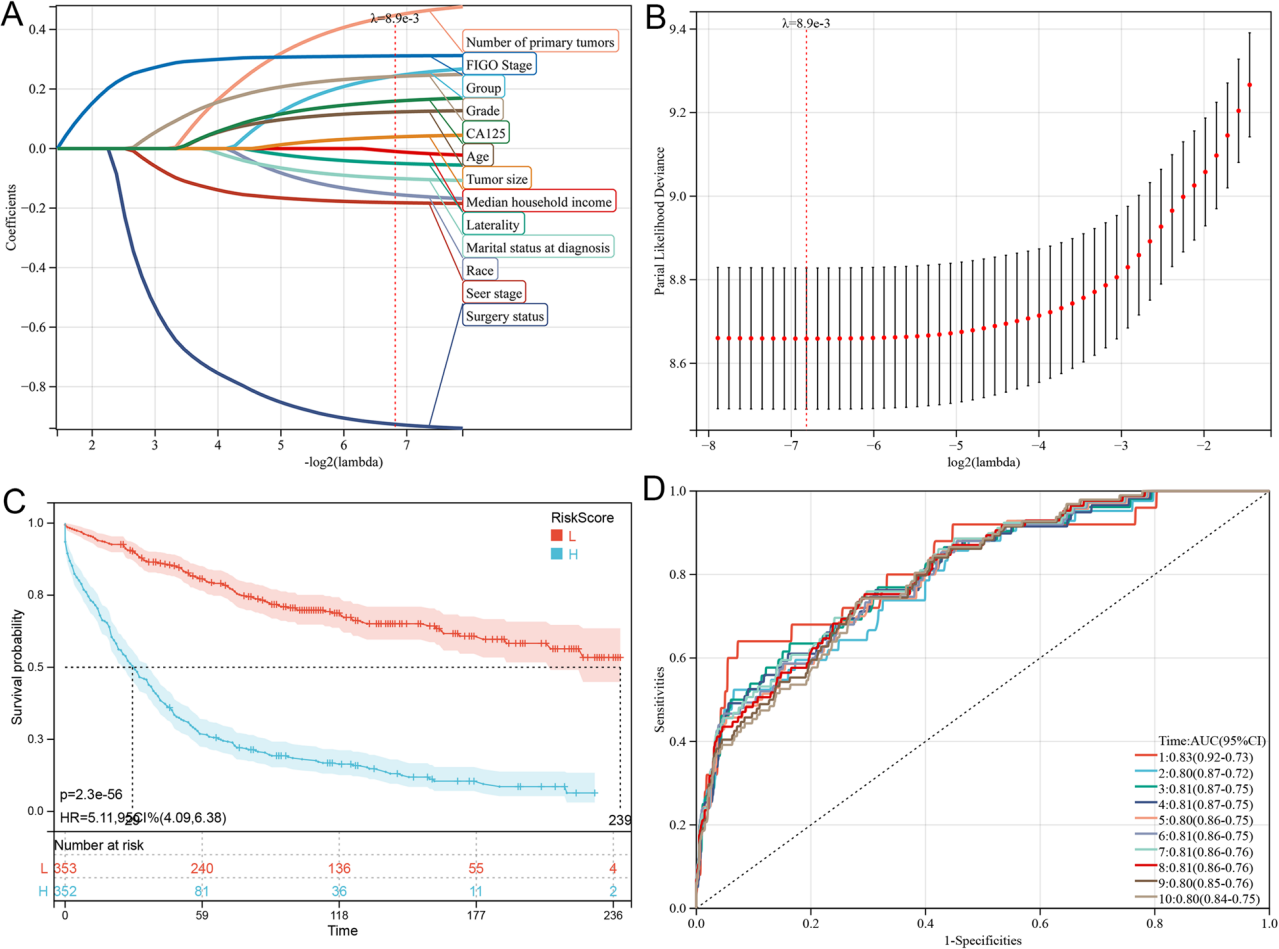
Construction and evaluation of overall survival-associated predictive models. **a** LASSO coefficient profiles and (**b**) LASSO deviance profiles depicting the optimal λ value and risk factors. **c** Kaplan–Meier survival curves of overall survival according to the risk scores; prognosis of the low-risk group is significantly better than that of the high-risk score group. **d** Receiver operating characteristic curves of overall survival at 1–10 years according to the risk scores in the predictive model data sets. LASSO, Least Absolute Shrinkage and Selection Operator

**Fig. 5:**
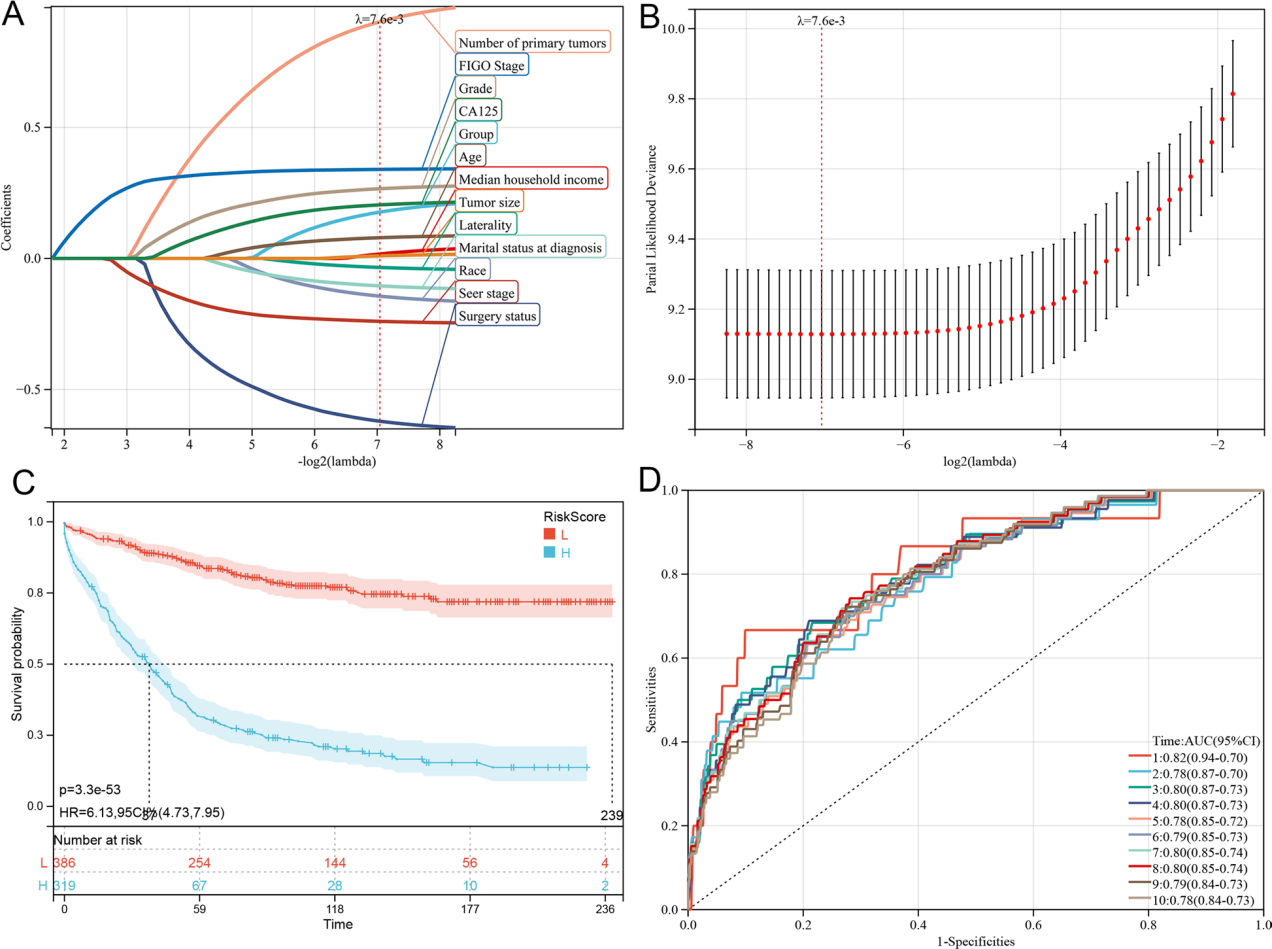
Construction and evaluation of cancer-specific survival-associated predictive models. **a** LASSO coefficient profiles and (**b**) LASSO deviance profiles depicting the optimal λ value and risk factors. **c** Kaplan–Meier survival curves of cancer-specific survival according to the risk scores; prognosis of the low-risk group is significantly better than that of the high-risk score group. **d** Receiver operating characteristic curves of cancer-specific survival at 1–10 years according to the risk scores in the predictive model data sets. LASSO, Least Absolute Shrinkage and Selection Operator.

Based on the analysis of the aforementioned prognostic factors, further study is warranted to determine whether the HGSOC and CENO groups have an impact on the prognosis of patients. Therefore, LASSO-Cox analysis was performed to construct predictive models of OS and CSS after univariate Cox analysis. The PP-Cox regression models of OS (Fig. 4a, b) or CSS (Fig. 5a, b) were constructed by integrating the significant prognostic factors in Table 3 and group information, respectively. After 10-fold cross-validation, the optimal λ values of 8.9e-3 and 7.6e-3 were obtained for the OS and CSS models, respectively. Finally, the group and 12 prognostic factors were determined in the predictive model of OS, comprising the number of primary tumors, FIGO stage, grade, CA125 status, age, median household income, tumor size, laterality, marital status at diagnosis, race, SEER stage, and surgery status (Fig. 4a). In the predictive model of OS, the risk score was generated using the following formula: Risk score = 0.244 × Group - 0.049 × Laterality - 0.926 × Surgery status + 0.447 × Number of primary tumors - 0.154 × Race - 0.100 × Marital status at diagnosis - 0.010 × Median household income + 0.123 × Age + 0.242 × Grade - 0.182 × SEER stage + 0.311 × FIGO stage + 0.039 × Tumor size + 0.159 × CA125 status. Coincidentally, the factors in the CSS predictive model were consistent with those in the OS model. Here, the risk score was generated using the following formula (Fig. 5a): Risk score = 0.177 × Group - 0.034 × Laterality - 0.620823597360226 × Surgery status + 0.903 × Number of primary tumors - 0.144 × Race - 0.105 × Marital status at diagnosis + 0.017 × Median household income + 0.079 × Age + 0.266 × Grade - 0.240 × SEER stage + 0.340 × FIGO stage + 0.010 × Tumor size + 0.204 × CA125.

Thereafter, data of 705 patients screened using PSM were subjected to survival analysis according to the risk score. The optimal cutoff value was determined to be 0.227 and 1.88 for OS and CSS, respectively. Subsequently, the included patients could be classified into high-risk or low-risk groups based on the cutoff value. The Kaplan–Meier curve analysis indicated that the predictive model of OS or CSS could distinguish patients with good or bad prognoses. The high-risk group exhibited a shorter OS than that of the low-risk group (Fig. 4c; *p* = 2.3e-56). Similarly, the high-risk group exhibited a shorter CSS than that of the low-risk group (Fig. 5c; *p* = 3.3e-53). Time-dependent receiver operating characteristic (ROC) analysis showed that AUC of risk score for OS prediction for 1–10 years were 0.83, 0.80, 0.81, 0.81, 0.80, 0.81, 0.81, 0.81, 0.80, and 0.80, respectively (Fig. 4d). In predictive model of CSS, the AUC value of the risk score for predicting the CSS for 1–10 years were 0.82, 0.78, 0.80, 0.80, 0.78, 0.79, 0.80, 0.80, 0.79, and 0.78, respectively (Fig. 5d).

**Fig. 6:**
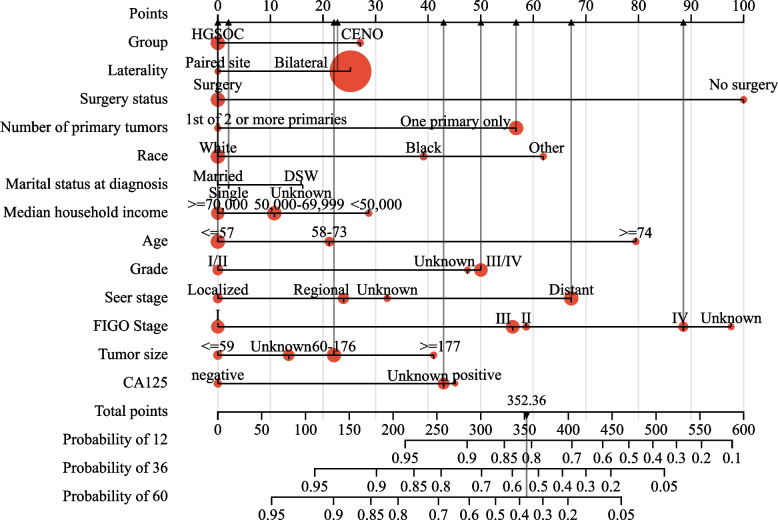
Nomogram of overall survival-associated predictive models. The larger the red dot, the greater the corresponding distribution frequency. The sum of the scores represented by the grey arrows represents the survival probability corresponding to 1, 3, and 5 years

**Fig. 7:**
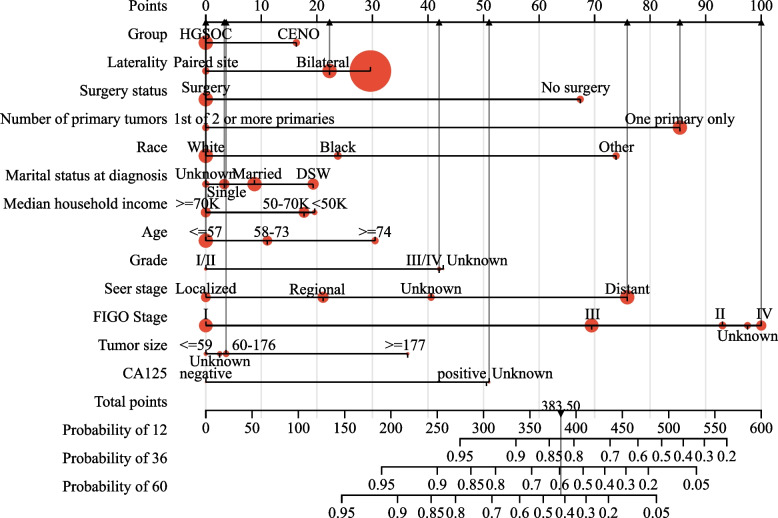
Nomogram of cancer-specific survival-associated predictive models. The larger the red dot, the greater the corresponding distribution frequency. The sum of the scores represented by the grey arrows represents the survival probability corresponding to 1, 3, and 5 years

The nomogram and calibration curve were applied in our study to illustrate the predictive model of OS (Fig. 6) and CSS (Fig. 7) more vividly and improve the practicality of this model. The nomogram included more than 13 traits, including the 12 prognostic factors, and group information. The score of each characteristic was determined by the scale on the top. The sum of the scores of the 13 traits was defined as the final score. We could estimate the prognosis of 1-, 3-, and 5-year OS and CSS for patients with ovarian cancer by the perpendicular line from the total point axis to the two-outcome axis.

## Discussion

In this study, we aimed to demonstrate the clinical features and prognosis of CENO based on the SEER database and explore its independent risk factors for OS and CSS. As HGSOC is the most common subtype of ovarian cancer clinically, we performed PSM between HGSOC and CENO to further clarify the malignancy of CENO after balancing the pathological features. As a rare, pathologically broad histotype in ovarian cancer, CENO comprises adenosquamous carcinoma and adenocarcinoma with metaplasia. It is mostly reported in the literature in the form of case reports and is characterized by adenocarcinoma with other components or cells transformed into other components [12, 18, 21]. HGSOC differs from other ovarian cancer histotypes because it is the most common of ovarian cancers with poor clinical outcomes and has a genetic predisposition in a large proportion of cases [22–24]. In this study, we collected the data of 216 patients with CENO and 31,567 patients with HGSOC from the SEER database. Our results showed that compared with HGSOC, CENO occurred in individuals generally younger than 57 years, more commonly in whites and singles, and was diagnosed more often in 2000–2001. The precise reasons for these differences remained unclear. At the time of diagnosis, CENO was unilaterally present in > 50% of the patients, with FIGO stage I/II, whereas HGSOC was bilaterally present in > 50% of the patients, with FIGO stage III/IV. This observation suggests that the natural progression of CENO may be slower than that of HGSOC. Paradoxically, patients with CENO were more likely to have a tumor size > 177 mm and a higher probability of positive lymph node metastasis at the time of diagnosis than patients with HGSOC. No statistical difference was observed between the two groups in terms of undergoing surgery; however, there was a difference in receiving radiotherapy (CENO: 6.0% vs. HGSOC: 0.8%) and chemotherapy (CENO: 65.7% vs. HGSOC: 79.4%). Patients with CENO are speculated to receive radiotherapy more often and receive chemotherapy less frequently than patients with HGSOC. Because HGSOC is a serous carcinoma, its CA125 positive rate in our study was high (61.1%), whereas that of CENO was only 11.1%, suggesting that serous CENO may account for a low rate in CENO [25].

Further, we explored the risk factors associated with CENO prognosis through univariate and multivariate regression analyses. Through univariate regression analysis, we found that age, race, marital status, median household income, CA125 status, surgery, tumor laterality, FIGO stage, SEER stage, and grade were associated with OS and CSS in patients with CENO. Notably, chemoradiotherapy was not related to the prognosis of patients with CENO, suggesting that surgery alone may be more beneficial for patients with CENO and also avoid the side effects of radiotherapy and chemotherapy. This hypothesis was verified by the multivariate regression analysis, where surgical treatment was an independent risk factor for OS and CSS in patients with CENO. Concurrently, the year of diagnosis, race, CA125 status, grade, and FIGO stage remained independent prognostic factors for patients with CENO, whereas tumor size, laterality, and patient age were not independent prognostic factors. This finding suggests that the therapeutic level of CENO has been improved to a certain extent in the past 10 years and that the prognosis of patients with serous CENO may be worse than that of patients with other types of CENO. Through the PSM method, the clinical characteristics between patients with HGSOC and patients with CENO were balanced, which was beneficial for comparing the clinical prognosis of these two pathological types. After matching, patients with CENO were observed to have worse OS and shorter median survival than patients with HGSOC (CENO: 53 months; HGSOC: 74 months). Moreover, we performed LASSO analysis and built predictive models for patiens with HGSOC and patients with CENO after PSM. These two models depicted the survival difference between patients with CENO and patients with HGSOC and will aid clinicians in judging the prognosis of patients with CENO.

Owing to its extremely low incidence rate, literature reports on CENO were limited to case reports and systematic reviews, in which the data were not sufficient to formulate a standard treatment plan. Sugiyama et al. [12] summarized nine cases of 32–57-year-old patients with ovarian adenosquamous carcinoma; the results of this study were consistent with our conclusion that patients with CENO were mostly younger than 57 years. Seven of the nine cases reported FIGO staging: five in stage I, one in stage III, and one in stage IV, which was also consistent with our conclusion that patients with CENO were mostly in stage I/II at the time of diagnosis. Five of the nine patients received postoperative chemotherapy with a median OS of 12 months (range: 3–66 months), and four of the five stage I patients experienced recurrence and died within 13 months. Combined with the results of this study, we could infer that although CENO patients were mostly diagnosed with stage I/II, their prognosis was not optimistic. In 2018, Yu et al. reported a 38-year-old woman diagnosed with stage II CENO with elevated preoperative CA125 levels [26]. This patient discontinued the drug regimen after receiving several cycles of postoperative chemotherapy and antiangiogenic therapy with no effect and significant side effects. Thereafter, she underwent the poly (adenosine diphosphate ribose) (PARP) polymerase and programmed death-1 (PD-1) inhibitor treatment; the OS was 15 months accompanied by remission. Based on our study findings, we inferred that surgery, not chemoradiotherapy, improves patient outcomes. In conjunction with this case report, we proposed that patients with CENO should be treated with drugs such as PARP and PD-1 inhibitors rather than chemoradiotherapy after surgery to achieve better treatment results. Lombard et al. summarized three cases of ovarian adenocarcinoma with squamous metaplasia [17]. These three patients were aged 46, 63, and 72 years, respectively; the former was in stage II at diagnosis, while the latter two were in stage III. The first patient did not respond to chemotherapy and had an OS of 24 months, and the second patient received chemoradiotherapy and had an OS of 48 months. Meanwhile, the third patient did not have any treatment records. AFP is a crucial feature of hepatocellular carcinoma, while hepatoid adenocarcinoma is a pathological type of ovarian cancer with a phenotype similar to that of hepatocellular carcinoma. Differentiation diagnosis of hepatoid yolk sac tumor is required to clinically distinguish ovarian cancer from hepatocellular carcinoma, which is defined by the expression of broad-spectrum cytokeratins, AFP, and hepatocellular antigens with the absence of sex cord and germ cell markers. Pins et al. summarized 27 cases of ovarian liver cancer reported on PubMed in 2019 [27]. These patients had a median age of 57 years (range: 35–78 years). Unilateral lesions were present in most of the patients at diagnosis (16 of 20, 80%), with elevated AFP levels (20 of 22, 90.9%), while metastases were rare at initial diagnosis. Most patients received surgery and chemotherapy treatment (16 of 17, 94.1%; 15 of 17, 88.2%), whereas only a few received radiotherapy (2 of 17, 11.8%). The median follow-up of this group was 10.5 months (range: 1–60 months). The majority of the patients with the aforementioned pathological types were not at an advanced stage when diagnosed, and most had unilateral lesions, received postoperative chemotherapy, and had a poor outcome. These results are consistent with those of our study.

To our knowledge, this is the first population-based study on CENO and a comparison between CENO and HGSOC. The clinical data of patients enrolled in our study included long-term follow-up records for up to 20 years, increasing the reliability of the analyses. Nevertheless, our study has some limitations. First, the data extracted from the SEER database did not specify the type of chemotherapy or the number of chemotherapy cycles received by the patient. Second, whether patients received targeted therapy such as PARP inhibitors was not specified in the dataset. Last, missing entries for some patients in the database may lead to biased analysis results. For example, it was difficult to explain why patients with unknown CA125 status had more favorable outcomes than those with negative CA125 status in the CENO group. Some unknown reasons or confounding factors might have led to this result. Therefore, we suggest that a global CENO database should be constructed for retrospective and prospective studies in order to develop appropriate treatment strategies.

## Conclusion

Owing to the extremely low incidence rate and lack of population-based research, no consensus treatment or guideline is currently available for CENO. To this end, we aimed to ascertain the clinical characteristics, treatment strategies, and prognosis of CENO based on a large-scale sample and compare them with those of HGSOC, the common subtype of ovarian cancer. We found that CENO was associated with decreased OS compared with HGSOC and determined the prognostic predictive models and nomograms to predict the individual survival rates of patients with CENO. These methods may assist clinicians to assess the risk of CENO among patients and make more advisable decisions regarding individual treatment.

## Materials and methods

### Patient selection

We screened the latest version of the SEER database, which was released in November 2021, using the SEER*Stat software (version 8.4.0.1). The Research Plus database, which includes all reportable cancer cases from 17 population-based cancer registries (2000–2019), was chosen for this study. Patients diagnosed with CENO or HGSOC between 2000 and 2017 were determined based on the SEER database information as follows: Site code: primary malignant tumor in the ovary; histology code: serous cystadenocarcinoma (8441/3), papillary serous cystadenocarcinoma (8460/3), and complex epithelial neoplasms (8560–8579/3) (8560/3: adenosquamous carcinoma; 8562/3: epithelial-myoepithelial carcinoma; 8570/3: adenocarcinoma with squamous metaplasia; 8574/3: adenocarcinoma with neuroendocrine differentiation; 8575/3: metaplastic carcinoma, NOS; 8576/3: hepatoid adenocarcinoma). The exclusion criteria were as follows: uncertain survival time or cause of death of the patients, CENO or HGSOC not being the first tumor, and unknown median household income. All patient data were downloaded through the SEER*Stat software (Fig. 1). Because the SEER database utilized in this article can be publicly accessed, informed consent and ethical review were not required for the analysis of patient data.

### Clinical characteristics and outcome measurement

The clinical characteristics identified in this study included the year of diagnosis, age, literality, surgery status, grade, tumor size, American Joint Committee on Cancer stage, median household income, SEER stage, chemotherapy status, marital status, OS, cancer antigen 125 (CA125) status, and vital status. Subsequently, the clinical characteristics were divided into the following categories: age at diagnosis (grouped into ≤57, 58–73, and ≥ 74 years), race (white, black, and other), marital status (single/unmarried, married, divorced/separated/widowed, and unknown), FIGO stage (I, II, III, IV, and unknown), surgery (yes and no), chemotherapy (yes, no, and unknown), radiotherapy (yes and no), laterality (bilateral, left, paired site, and no information concerning laterality and right), number of primary tumors (one primary tumor and first of ≥2 primary tumors), year of diagnosis (2000–2001, 2002–2005, and 2006–2017), grade (I/II, III/IV, and unknown), lymph node status (negative, positive, and unknown), tumor size (≤59, 60–176, and ≥ 177 mm), and CA125 status (negative, positive, and unknown).

The first endpoint measurement was OS, which was calculated as the time interval from the diagnosis of ovarian cancer to death from any cause. The second endpoint measurement was CSS, which was calculated as the time interval from the diagnosis of ovarian cancer to death from the same disease. The final follow-up date was December 31, 2019.

### Statistical analysis

Chi-squared or Fisher’s exact test was used to compare the clinical characteristics of patients with CENO and those with HGSOC. Each CENO case was matched with four HGSOC case through the propensity score matching (PSM) method using the R package “MatchIt” 16 version 4.1.0 with a caliper of 0.05. All clinical characteristics were included. Kaplan–Meier curves were used for comparing the OS and CSS rates of the CENO and HGSOC groups before and after PSM. The factors potentially influencing the OS or CSS of patients with CENO were analyzed using univariate and multivariate Cox regression analysis, and the log-rank test and Breslow test were utilized for comparison in Kaplan–Meier analysis.

The Least Absolute Shrinkage and Selection Operator (LASSO) regression analysis was used to identify the best weighting coefficient of clinical characteristics in predicting the prognosis of patients with CENO or HGSOC after PSM. LASSO is a modification of least squares utilized in penalty terms and regularization methods for statistical modeling and suppressing overfitting. Optimal values for the lambda parameters (λ = 0.012 in OS and λ = 0.019 in CSS) were found by a tenfold cross-validation using the cv.glmnet function of the ‘glmnet’ package in R.

Further, we used the ‘pROC’ (version 1.17.0.1) package in R to perform a receiver operating characteristic (ROC) analysis of the follow-up time and LASSO risk scores of patients from 1 to 10 years. Moreover, the area under the curve (AUC) and confidence intervals were evaluated. Based on the best cut-off value calculated using the ‘maxstat’ (version: 0.7–25) package or median of risk scores, we divided the patients into two groups with high and low risk and further used the survfit function of the ‘survival’ package to analyze the difference in prognosis between these groups. The log-rank test method was employed to evaluate the significance of prognostic differences between different groups of samples.

Finally, using the ‘rms’ package in R, we integrated the data of survival time, survival status, and significant characteristics in LASSO analysis to build a nomogram by using the Cox method and assess the prognostic significance of these characteristics in 705 patients with CENO or HGSOC after PSM. By integrating multiple predictors and drawing multiple lines with scales in proportion, the nomogram can easily calculate the risk of disease or the probability of survival of an individual. The C-index was used to evaluate the efficacy of the nomogram. Additionally, the prognosis of patients with different pathological types was compared, and PSM analysis of clinical characteristics was performed to reduce confounding bias.

All data were analyzed using the R software (version 4.2.1), and *p*-values < 0.05 were considered to be statistically significant.

## Data Availability

The datasets generated and/or analyzed during the current study are available in the Surveillance, Epidemiology, and End Results database, [https://seer.cancer.gov/].

## Abbreviations

CENO: Complex epithelial neoplasms of the ovary
HGSOC: High-grade serous ovarian cancer
SEER database: the Surveillance, Epidemiology, and End Results database
AFP: Alpha-fetoprotein
OS: Overall survival
CSS: Cancer-specific survival
LASSO: Least Absolute Shrinkage and Selection Operator
PSM: Propensity score matching
CA125: Cancer antigen 125
ROC: Receiver operating characteristic
